# Supplementary figures and images for: TNF licenses macrophages to undergo rapid caspase-1, -11, and -8-mediated cell death that restricts Legionella pneumophila infection
Source: PLoS Pathog. 2023 Jun 6;19(6):e1010767. doi: 10.1371/journal.ppat.1010767 (PMC10275475; doi:10.1371/journal.ppat.1010767)

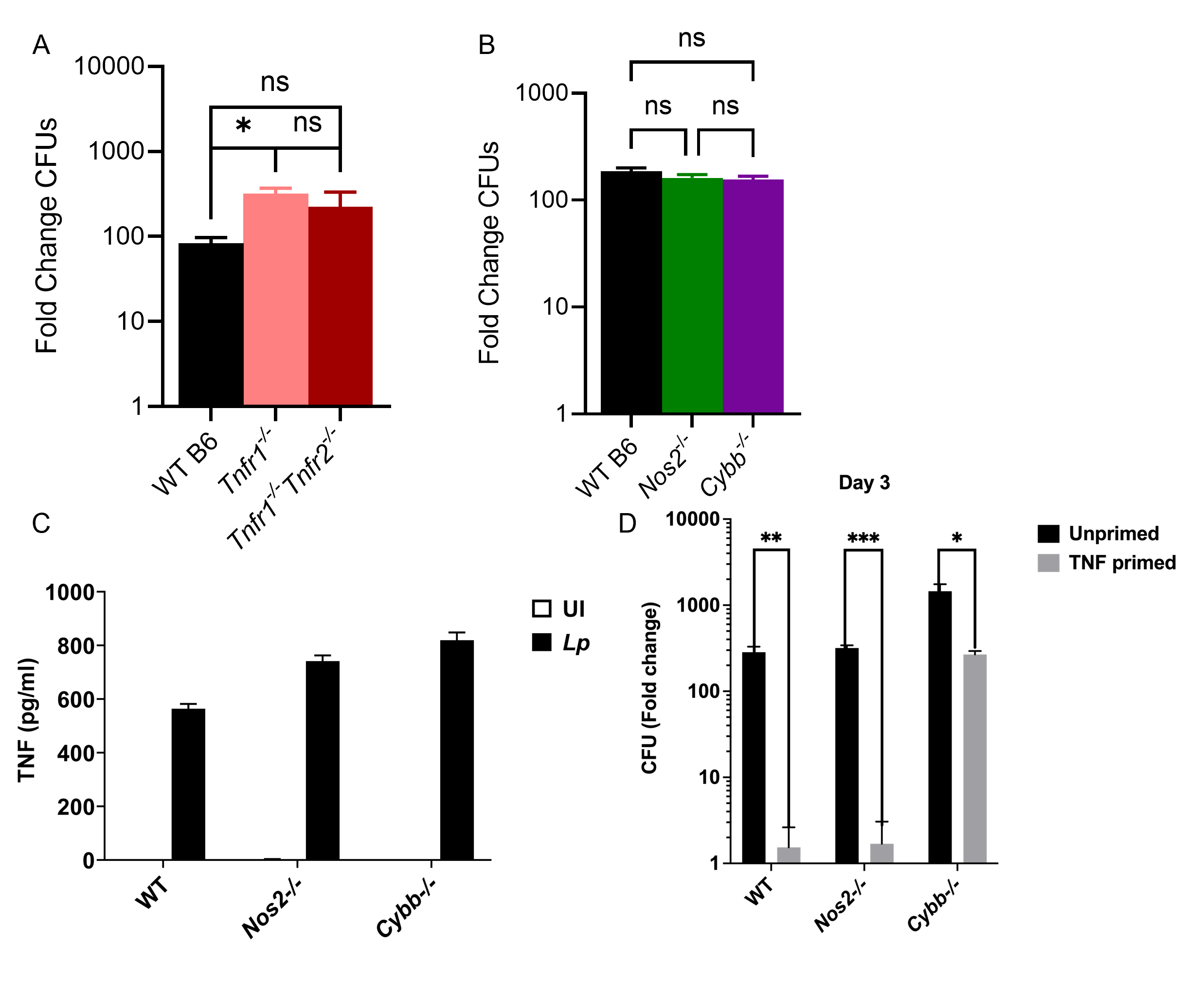

Supplement: S1 Fig — (A) WT, Tnfr1-/-, and Tnfr1-/-Tnfr2-/- BMDMs were infected with replicating ΔflaA L. pneumophila at MOI = 1. The fold change in CFUs was quantified at 72 hours post-infection. (B-D) WT, Nos2-/-, and Cybb-/- BMDMs were infected with replicating (B, D) or non-replicating (C) ΔflaA L. pneumophila at MOI = 1 (B,D) or 10 (C). The fold change in CFUs was quantified at 72 hours post-infection, while TNF secretion was assessed at 16 hours post-infection. * is p<0.05 ** is p<0.01, and *** is p<0.001 by one-way ANOVA with Tukey HSD post-test (A,B) or Student’s t-test (D), ns is not significant. (TIF) [file ppat.1010767.s001.tif]

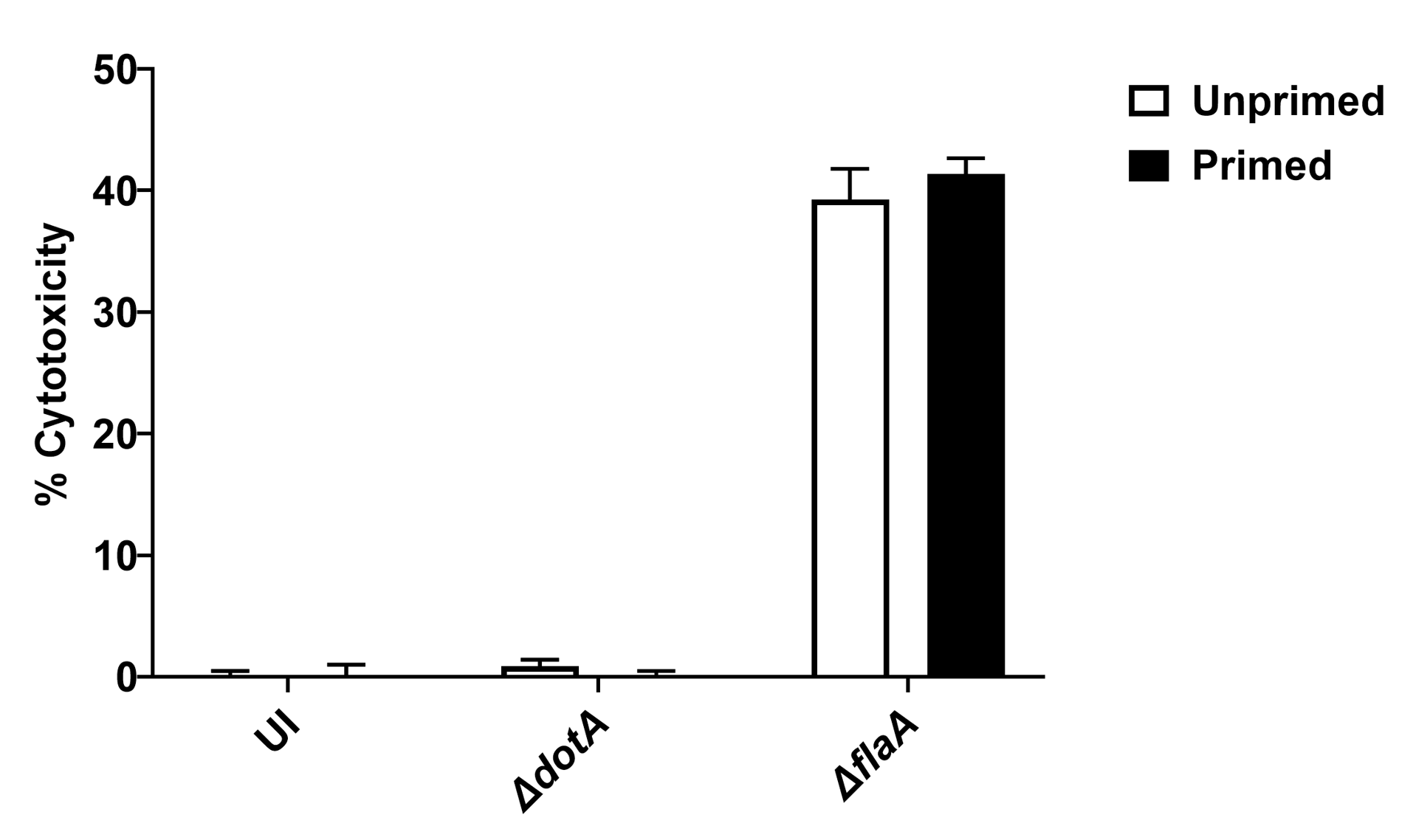

Supplement: S2 Fig — WT BMDMs were uninfected or infected with non-replicating L. pneumophila ΔflaA or ΔdotA mutant strains at MOI = 10 for 16 hours. Cells were primed with either 10 ng/mL rTNF or PBS mock control for 16 hours prior to infection. (TIF) [file ppat.1010767.s002.tif]

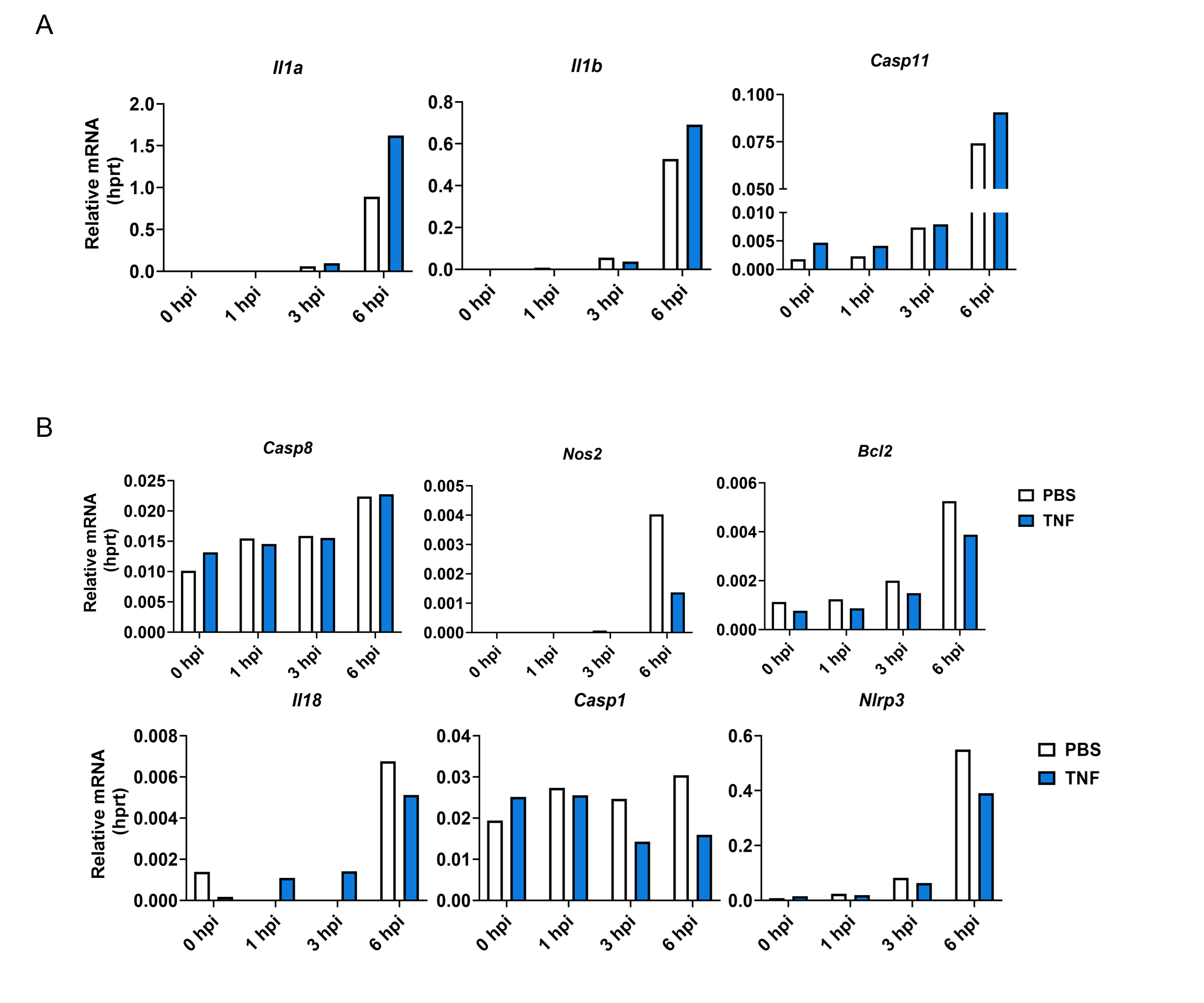

Supplement: S3 Fig — WT cells were primed with either 10 ng/mL rTNF or PBS for 16 hours, then infected with non-replicating ΔflaA L. pneumophila at MOI = 50. Supernatants and cell lysates were collected for immunoblot and RT-qPCR analysis. (TIF) [file ppat.1010767.s003.tif]

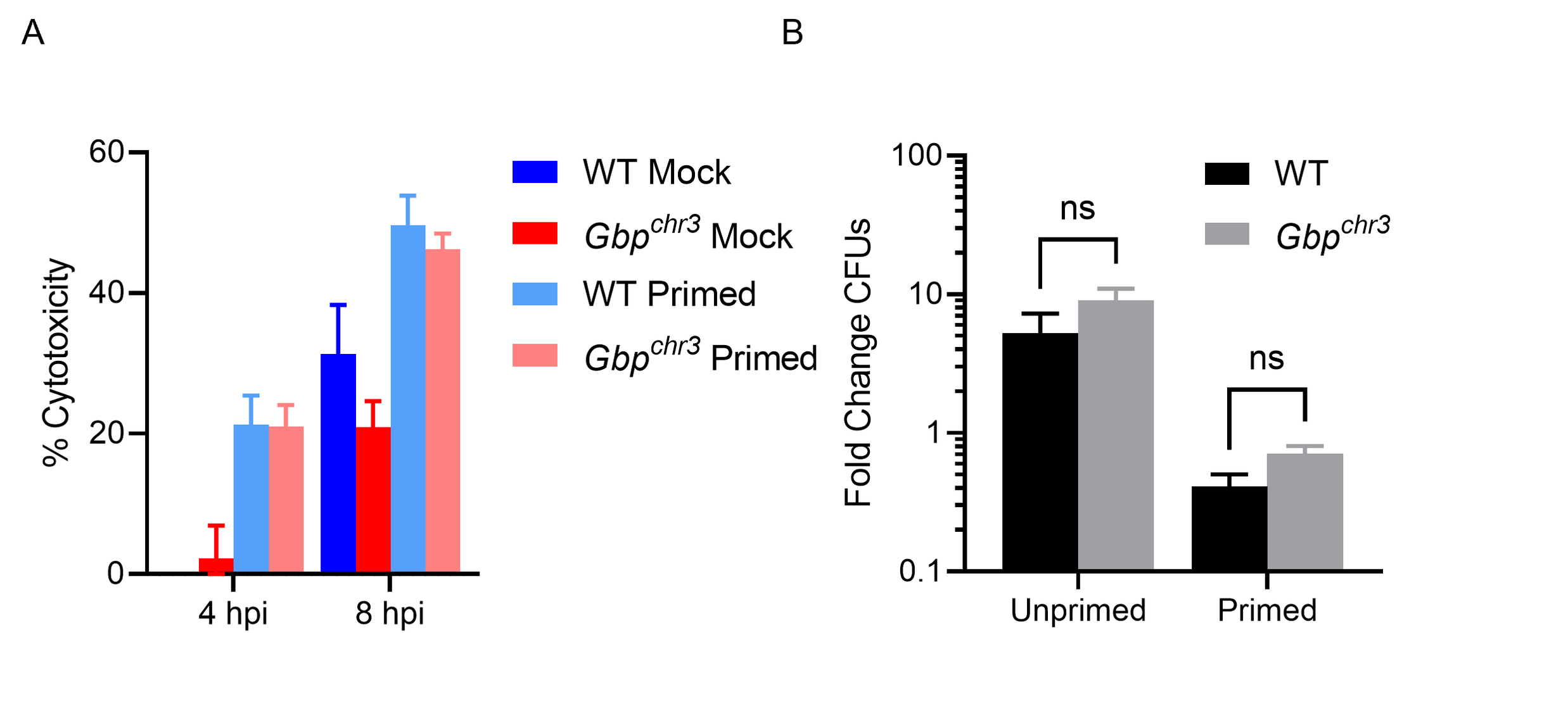

Supplement: S4 Fig — WT and GBPchr3 BMDMs were primed with either 10 ng/mL rTNF or PBS mock control for 16 hours prior to infection with non-replicating (A) or replicating (B) ΔflaA L. pneumophila at MOI = 10 (A) or 1 (B). (A) Cytotoxicity was measured by LDH release assay. (B) The fold change in CFUs was determined at 48 hours post-infection. Data shown are representative of two independent experiments. (TIF) [file ppat.1010767.s004.tif]

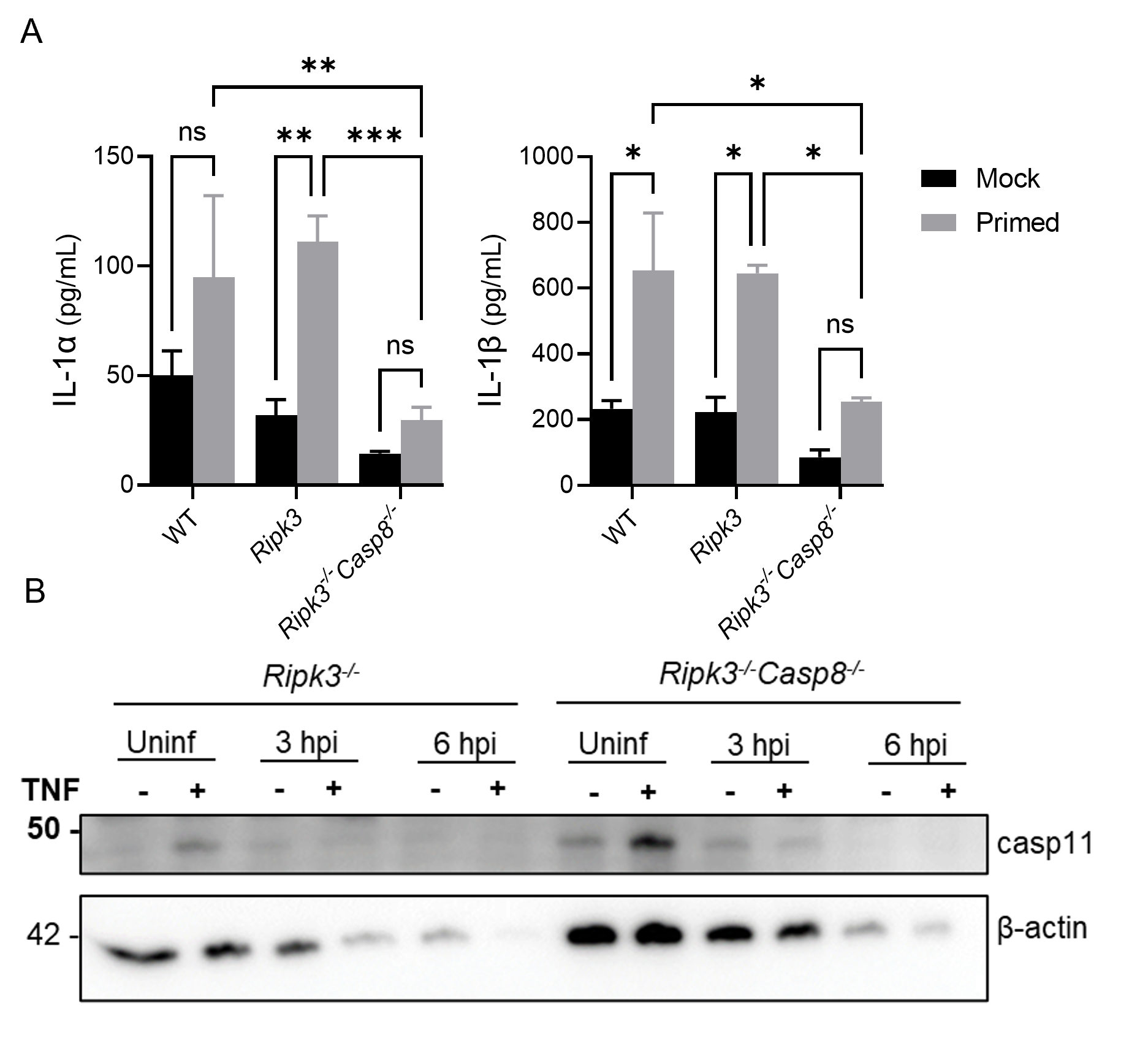

Supplement: S5 Fig — Ripk3-/- and Ripk3-/-Casp8-/- BMDMs were primed with either 10 ng/mL rTNF or PBS mock control for 16 hours prior to infection with non-replicating ΔflaA L. pneumophila at MOI = 50. (A) Supernatants were analyzed by ELISA at 24 hpi. (B) BMDMs were lysed and analyzed by immunoblot for caspase-11 protein at indicated timepoints. Graphs show the mean ± SEM of triplicate wells. * is p<0.05, ** is p<0.01, and *** is p<0.001 by 2-way ANOVA with Tukey HSD post-test. (TIF) [file ppat.1010767.s005.tif]

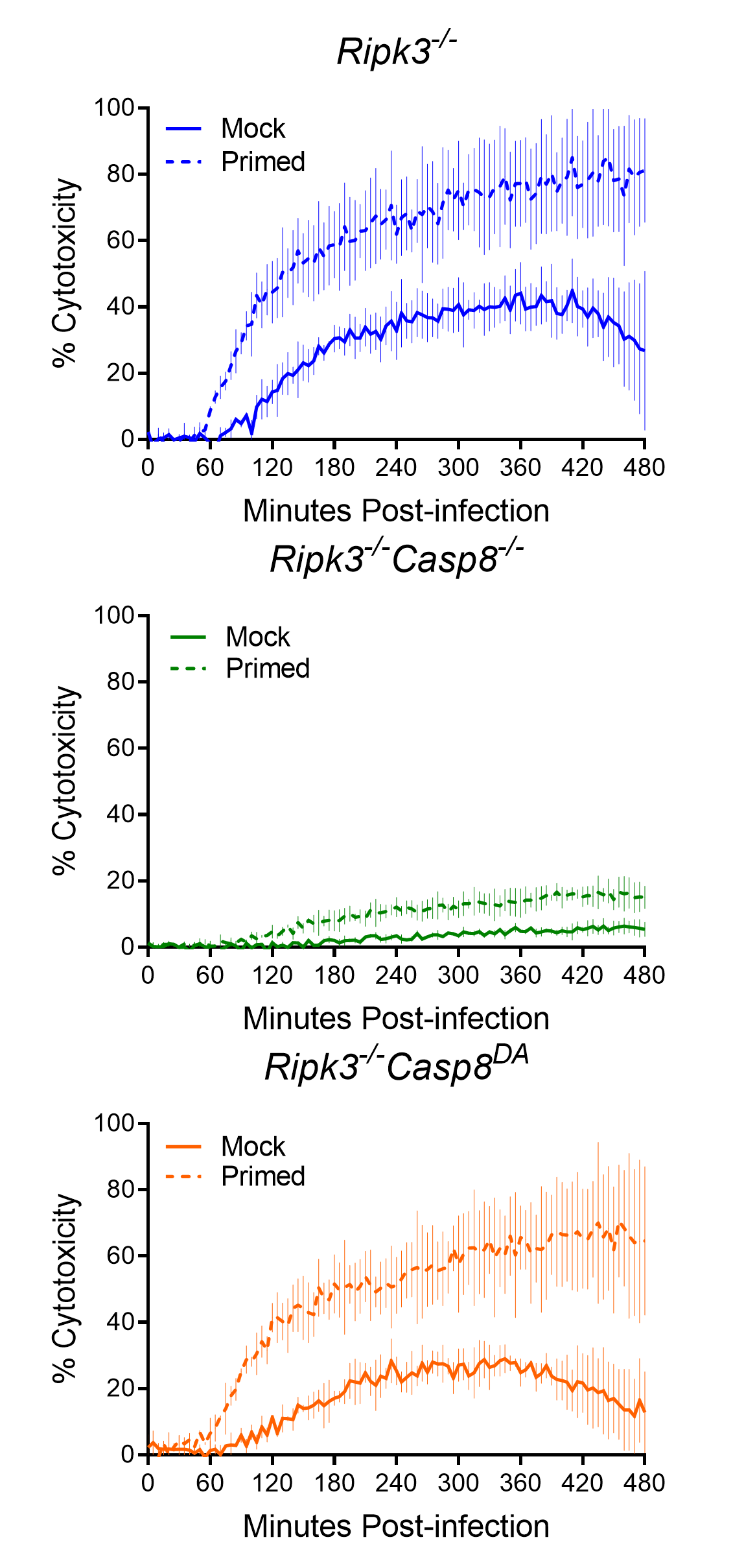

Supplement: S6 Fig — Ripk3-/-, Ripk3-/-Casp8-/-, and Ripk3-/-Casp8DA cells were infected with non-replicating L. pneumophila ΔflaA mutant strain at MOI = 10 for 8 hours. Cells were primed with either 10 ng/mL rTNF or PBS mock control for 16 hours prior to infection. (TIF) [file ppat.1010767.s006.tif]
