# Supplementary material for: TNF licenses macrophages to undergo rapid caspase-1, -11, and -8-mediated cell death that restricts Legionella pneumophila infection
Source: PLoS Pathog. 2023 Jun 6;19(6):e1010767. doi: 10.1371/journal.ppat.1010767 (PMC10275475; doi:10.1371/journal.ppat.1010767)
Supplement: Attachment — Submitted filename: TP_SS_ResponseLetter.docx [file ppat.1010767.s007.docx]

**Response to Reviewers**

We would like to thank the reviewers for their helpful comments on our work, and the opportunity to submit a revised version of our manuscript entitled “TNF licenses macrophages to undergo rapid caspase-1, -11, and -8-mediated cell death that restricts *Legionella pneumophila* infection.” The reviewers suggested a number of experiments which we have now performed and which we believe have strengthened our manuscript and its conclusions. In accordance with reviewer comments, we have also expanded discussion of our findings in the broader context of the cell death biology field, as well as modified the text in relation to our proposed mechanism and some of the methods for the sake of clarity. Below, we provide a point-by-point response to the reviewer’s comments. For clarity, each reviewer comment is italicized and followed by our response. Thank you once again for considering our manuscript for publication in *PLoS Pathogens*.

**Reviewer #1:**

Overall Comments: *In this study by Pollock et al., the authors address the role of TNF in mediating control of intracellular Legionella by pathways that lead to death of infected macrophages. The manuscript is very well-written and the data are convincing that at least one major mechanism by which TNF contributes to host defense against Legionella is licensing macrophages to undergo rapid caspase-1, -11, and -8-mediated macrophage death. In addition, they show that TNFR1 signaling is required for restriction of Legionella infection of macrophages and primes cells to rapidly undergo cell death. They go on to demonstrate that the T4SS is somehow triggering caspase-1 and -11 activation in TNF-primed macrophages and that TNF upregulated the expression of IL1a, IL1b, and IL18 mRNA as well as the expression of Caspase-11 protein. They also show that in macrophages from GbpChr3-/- mice that cell death and bacterial control were not abrogated. These findings suggest that TNF-dependent upregulation of inflammasome components leads to rapid engagement of non-canonical inflammasome activation independent of GBPs on chromosome 3. Finally, in a series of elegant experiments utilizing various BMDMs from KO and transgenic mice that caspase-8 contributes to cell death independently of its autocleavage downstream of TNF signaling. Finally, they demonstrate that TNR1 and caspse-8 are required for control of pulmonary Legionella infection in a mouse model. The manuscript is excellent and the findings are a significant contribution to the field and I have only minor suggestions to address before publication in Plos Pathogens.*

**Response:** We are grateful to the reviewer for their constructive suggestions and positive comments on our work.

Minor Comment #1: *The authors show that triggering cell death pathways in macrophages is dependent on the T4SS. Can they speculate what the host might be sensing that then leads to activation of caspases-1 and -11 and finally caspase-8? What is upstream that is dependent on TNF, but independent of GBPs on chrom3?*

**Response:** We thank the reviewer for this comment and have accordingly edited the discussion to include speculation as to the T4SS-mediated signal detected by host macrophages. I brief, we hypothesize that either an uncharacterized *Legionella* effector or the process of effector injection itself triggers NLRP3 activation in the context of TNF priming.

Minor Comment #2: *In Figure 6, is it possible to look in infected lungs and quantitate macrophage death to see differences in WT compared to KO mice? And does this correlate with differences in influx of cells that can kill Legionella e.g., perhaps higher levels of neutrophils in WT compared to KO’s?*

**Response:** We thank the reviewer for this insightful comment. We agree that it would be of interest to quantitate macrophage death in the lungs during infection, especially in the context of TNF signaling or deficiency. However, we have found from our own experience that quantifying macrophage death during *in vivo* infection of the lung is very challenging with the current experimental tools available to the field. We are currently developing tools to address this question in future studies.

**Reviewer #2:**

Overall Comments: *In this manuscript, Pollock et al demonstrate a novel role for TNF in caspase-mediated cell death in response to Legionella pneumophila infection. It is clear that TNF is important for pulmonary control of L. pneumophila, and the data presented reveal that caspase-1, -11, and 8 are essential for NAIP5/NLRC4-independent TNF-mediated restriction. The manuscript is well-written and the data are rigorous. I have some comments on interpretation of the data and further discussion of these data in the context of the published literature, which will further support/refine the model. Overall, this work is an important contribution to the field of antimicrobial innate immunity and host response to intracellular bacterial pathogens.*

**Response:** We are grateful to the reviewer for their thoughtful suggestions and positive comments about our work.

Major Comment #1: *Lines 144-154: These data do not rule out a role for ROS since mtROS are not evaluated (and are upregulated downstream of TNF signaling). To support this conclusion, additional data are required (antioxidant treatment) or the text should be modified to specify NOX2-mediated ROS (and not total ROS). Is the quantity of TNF secreted consistent between wt and Nos2-/- and cybb-/- BMDMs? Do iNOS or NOX2 contribute to bacterial restriction in TNF-primed BMDMs? The authors should also consider discussing their work in the context of the study by Roca et al (PMID: 31474371) showing a role for mtROS in TNF-mediated mediated cell death and restriction of Mycobacterium spp.*

**Response:** We would like to thank the reviewer for this helpful feedback regarding the role of ROS in TNF-mediated control of *L. pneumophila* infection. We have performed the following:

1. Modified the text to specify that we have only assessed the contribution of NADPH Oxidase-derived ROS, not mitochondrial ROS. We also discuss our work in the context of the Roca et al study.
2. We have performed additional experiments and added new figures (Figure S1C-D) examining the potential role of CYBB and iNOS in promoting TNF secretion and TNF-dependent bacterial restriction. Interestingly, our new data demonstrate that CYBB, while dispensable for control of *Legionella* growth in the absence of TNF signaling, partially contributes to the TNF-mediated restriction of replication we observe. We present the new data in a new Figure S1D.

Major Comment #2: *It's difficult to compare cell death between Ripk3-/-Casp8DA, Ripk3-/-, and Ripk3-/-Casp8-/- since they are plotted on separate histograms. Plotting all of the PI-uptake data on the same histogram would be a mess, so showing LDH data here would be more intuitive for comparison between these BMDMs. The authors could consider including the PI uptake data as supplemental information.*

**Response:** We thank the reviewer for this helpful comment. We have thus simplified Figure 4C to include only the cell death levels from primed cell populations. As per the reviewer’s suggestion, we have additionally included the original comparison of primed and unprimed cells in the Supplemental Figure S5.

Major Comment #3: *Lines 312-315/Fig 4D: Could decreased Casp3/7 activity in primed cells be a consequence of less viable cells? I'm not sure how well this assay detects enzymatic activity in cell supernatants. Can the authors comment on this? Perhaps it would be worth normalizing these data to viable cells?*

**Response:** We thank the reviewer for this comment. The Caspase-GLO assay assesses combined whole cell lysates and cellular supernatants for the presence of active caspases with specificity for substrate reporter molecules. All wells were seeded with equivalent cell density, and thus we find it likely that the decreased caspase-3/7 activity is, in fact, due to a loss of viable cells. If the caspases were active prior to loss of cell viability, they would still present with activity in the GLO assay, as their activity would still be measured in the mixed lysate/supernatant sample preparation.

Major Comment #4: *Lines 344-345: It seems that gasdermin-dependent cell death is not responsible for L. pneumophila restriction. How do the authors reconcile this with their overall model whereby TNF-mediated cell death is responsible for bacterial restriction? It would seem that the 'additional TNF-mediated protective mechanisms' are quite important here. In Fig 5B, it seems that there is some gasdermin-independent cell death at 8h post-infection. I suggest modifying lines 357-359 to address this. Based on the work of Oxenius and colleagues, it may be phagolysosomal fusion (PMID: 27105352). It would be interesting to see whether this is the case, but I recognize it is likely out of the scope of the study. Please provide additional discussion on potential alternative mechanisms of bacterial restriction.*

**Response:** We would like to thank the reviewer for this suggestion. We agree that gasdermin-dependent cell-death does not appear to be necessary for TNF-mediated restriction of *L. pneumophila* infection, suggesting that there are other TNF-dependent mechanisms that contribute to the restriction of *L. pneumophila* in the absence of gasdermin-mediated cell death. We would like to note that, in the absence of gasdermin D and E, we still observe TNF-licensed cell death at later timepoints post-infection, as presented in Figure 5B. These data indicate that there are other cell death pathways that compensate in the absence of gasdermins and may contribute to TNF-mediated restriction of *L. pneumophila*. We additionally appreciate the recognition that further exploration of these additional mechanisms is outside the scope of the current study, which we intend to investigate in a future study. In the meantime, we have modified language in this manuscript as suggested, as well as provided additional discussion of further TNF-dependent mechanisms of restriction that are independent of gasdermins, including phagolysosomal fusion.

Minor Comment #1: *Lines 180-182: It seems that only PI uptake assays are shown. Please also include the LDH-based cytotoxicity data.*

**Response:** We thank the reviewer for this suggestion. We have now included the LDH-based cytotoxicity data as a supplemental figure (Fig S2).

Minor Comment #2: *The statement '… other caspase-1/11-independent factors contribute to TNF-mediated cell death…' is confusing in light of the data in Fig 2B-C and the statement in lines 188-190. Can the authors please clarify what the statement is based on?*

**Response:** We posit that additional caspase-1/11-independent factors contribute to death as we observe that, in the absence of caspases-1 and -11, there is delayed death as detected by PI uptake in Fig. 2C. Based on our other data presented in this manuscript, we believe that this caspase-1/11-independent death is caspase-8 dependent.

Minor Comment #3: *Is there evidence that these GBPs are actually present in infected BMDMs in the absence of IFN-g priming?*

**Response:** We thank the reviewer for this question. It was previously shown in the doctoral thesis of Dr. William Bradley, a former graduate student in the Shin lab, that mRNA transcripts of murine *Gbp1* and *Gbp2* (Fig. R1) are induced in BMDMs during TNF priming and in the absence of IFN-γ.

Minor Comment #4: *Figure 4D: It would be helpful to make the Tnf-/- primed bars a different color since it looks a lot like the red used for Tnf-/- mock.*

**Figure 1. TNF priming upregulates GBP mRNA expression.** C57BL/6 BMDMs were treated with vehicle control or 10 ng/mL recombinant murine TNF for 16 hours. Fold-induction of mGBP-1 and mGBP-2 was measured via qRT-PCR. Graphs show the mean ± SEM of triplicate wells. Representative of two independent experiments.

**Response:** We thank the reviewer for this suggestion. We have adjusted the color of the primed bars to differentiate them from unprimed samples, as suggested.

Minor Comment #5: *Figure 5B. Please comment on gasdermin-independent cell death observed at 8h post-infection in TNF-primed cells*

**Response:** We thank the reviewer for this suggestion. We comment on this finding and have added additional text to both the results and conclusion sections outlining the mechanisms of redundancy we believe to be at work in TNF-licensed but gasdermin-independent cell death.

Minor Comment #6: *Lines 343-345: The data in Fig 5D suggest to me that bacterial restriction is independent of cell death*

**Response:** The data in Fig. 5D certainly suggest that bacterial restriction does not depend on gasdermin-dependent pyroptosis – however the data in figure 5B additionally suggest that in the absence of gasdermins, there is still some death during infection. Preliminary data suggest that caspases-3 and -7 are catalytically active in TNF-primed cells only in the absence of gasdermins. We thus propose that a delayed death pathway may compensate at later times post-infection in the absence of gasdermins. This is the subject of ongoing research, and we intend to address the mechanism of this cell death in future studies.

Minor Comment #7: *Lines 460-462: What do the authors think the pro-inflammatory genes could be?*

**Response:** We thank the reviewer for this question. Based on data from the Brodsky, Dixit, and Newton groups, a number of pro-inflammatory cytokine genes are modulated by caspase-8, including *Il12, Il1a, Il1b, Il4i1* [1, 2]. We have expanded this portion of the text to include this relevant information.

Minor Comment #8: *Line 509: What was the MOI used for Fig 1H?*

**Response:** We thank the reviewer for drawing attention to information missing from Figure 1’s legend. We have updated the figure legend to include the MOI used in panel H.

**Reviewer #3:**

Overall Comments: *This is a comprehensive study focused or the role of TNF licensing in mediating macrophage death in response to L. pneumophila. The authors demonstrated that TNF priming triggers cell death, however, the downstream mechanisms remain unclear. They show that TNF signaling licenses macrophages to die rapidly in response to Legionella infection mainly by rapid gasdermin-dependent, pyroptotic death downstream of inflammasome activation. They also show that TNF signaling upregulates components of the inflammasome response. They suggest that caspase-11 is rapidly activated, while caspase-1 and caspase-8 mediate delayed pyroptotic death. Furthermore, caspase-8 is required for control of pulmonary Legionella infection. Together, they propose that all three caspases are collectively required for optimal TNF-mediated restriction of L. pneumophila replication in macrophages. These findings reveal a TNF-dependent mechanism in bone marrow derived macrophages for the activation of rapid cell death that is collectively mediated by caspases-1, -8, and -11 and subsequent restriction of Legionella infection. While the findings are mechanistically important, the authors need to address some points.*

**Response:** We are grateful to the reviewer for their thoughtful suggestions and positive comments about the mechanistic importance of our work

Major Comment #1: *The authors did not examine other specific forms of cell death including PANoptosis and the downstream repercussions. LDH release and PI uptake can occur in response to several forms of cell death.*

**Response:** While LDH release and PI uptake occur in response to several forms of cell death, our findings robustly demonstrate a requirement for caspases-1, -8, and -11 and the pyroptotic mediators gasdermins D and E in TNF-licensed cell death early during *L. pneumophila* infection, while observing no role for RIPK3 (essential for necroptosis and PANoptosis) nor the auto-processing activity of caspase-8 (required for classical extrinsic apoptosis.) We additionally observed no caspase-3/7 activity, which is an indicator of and required step for apoptosis. Based on the functional requirement for caspases-1, -8, and -11 and GSDMD/E, and no detectable requirement for RIPK3 or activation of caspases-3 and -7, our data indicate that the nature of this TNF-licensed death is predominantly pyroptotic.

Major Comment #2: *The authors mention in the legends of several figures using non-replicating fla mutants. They did not explain the characteristic of this unusual fla mutant. Typically, Legionella fla mutants replicate very well even in restrictive cells.*

**Response:** We thank the reviewer for noting this point. The flagellin-deficient mutants used in this experiment are from the LP02 strain background, which is a thymidine auxotroph and thus incapable of replication in absence of exogenous thymidine supplementation. We used this strain to avoid the potentially confounding effect of bacterial replication when measuring cell death in infected macrophages. We have clarified this language in the text to avoid any further confusion.

Major Comment #3: *Please add the name of the different mutants above every panel. It is confusing to follow which ones included WT legionella, DotA mutant or Fla mutant.*

**Response:** We thank the reviewer for this suggestion. Every experiment included in this manuscript made exclusive use of the Δ*flaA* mutant strain of bacteria, except for Figure 2A, which used a T4SS mutant. Thus, we have instead opted to more clearly and carefully present in the text the nature of our choice of bacterial strains.

Major Comment #4: *In several figures, they focus on IL-1a and not IL-1B. Although IL-1B is the well characterized inflammasome-dependent cytokine whereas IL-1a is not.*

**Response:** We assessed both IL-1α and IL-1β levels in most of the manuscript. We and other groups have previously found that IL-1α tracks well with IL-1β in terms of being an inflammasome-dependent cytokine.

Major Comment #5: *The authors conclude that TNF licenses macrophages to undergo caspase-1 and 11-mediated cell death to restrict Legionella, yet in figure 2 E, primed Casp-1-/-/casp11-/- macrophages still restrict Legionella replication.*

**Response:** We conclude in this manuscript that TNF-licensed cell death occurs via caspase-1/11 as well as caspase-8 following infection. In figure 5, we demonstrate that while caspases-1/, -11 and -8 are individually dispensable for restriction of *Legionella* infection, in the absence of all three caspases, *L. pneumophila* is able to replicate even in TNF-primed BMDMs.

Major Comment #6: *The authors consider the increase of caspase-11 expression as a sign of activation, which is incorrect. They also use the cleaved caspase-11 band on western blots as a sign of activation, which is also incorrect, since it is known in the field that cleavage of caspase-11 does not signify activation. Instead, they need to use activation assays specific to caspase-11 as published by others.*

**Response:** We thank the reviewer for this comment. We have made sure to clarify in the text when referring to increased expression of caspase-11 rather than activation. As for immunoblot-independent assays of caspase-11, while it is true that cleavage of caspase-11 does not necessarily indicate active processing of caspase-11 substrates, it has been well established in the field that caspase-11 cleavage correlates with activation [3-7]. We are unaware of any caspase-11-specific activity assays currently available commercially. If the reviewer is referring to Ross et al 2018 [8]**,** this study used an LEHD substrate and referred to it as ‘caspase-11-*like* activity,’ as this substrate is classically used to detect caspase-9 activity. While it may be that such an assay might work in a purified system or cell-based overexpression setting, during bacterial infection of macrophages this assay is unlikely to provide information about caspase-11-specific activity.

Major Comment #7: *It is not clear why caspase-11 cleavage products are detected in culture supernatants and not in cell lysates (Fig. 3G).*

**Response:** We think that caspase-11 cleavage products are predominantly detected in cell supernatants as cells that harbor active caspase-11 undergo pyroptosis and lose plasma membrane integrity.

Major Comment #8: *There is a difference between the secretion of cytokines and the passive release of cytokines from dying cells. The authors need to accurately state these differences and use them appropriately through the text while explaining their results.*

**Response:** We thank the reviewer for this suggestion. We have modified the text to more clearly refer to release as opposed to secretion.

Major Comment #9: *SFig 4B is confusing. Why is there no actin in the cell lysates? What is the band that they show as casp11? Why does it disappear when actin disappears?*

**Response:** We hypothesize that both actin and caspase-11 are lost from the cellular lysate when the molecules are released into the sample supernatant, as we observe in Figure 3G, during pyroptotic cell death.

Major Comment #10: *The authors need to explain how can “uncleavable” caspase-8 modulate Legionella replication?*

**Response:** We clarify that the *Casp8^DA^* mutant used in this manuscript is still capable of being cleaved – it is only incapable of engaging in autocleavage to stabilize itself in a homodimer. Other studies have demonstrated that caspase-8 autocleavage is dispensable for many non-apoptotic effects of caspase-8 signaling [9, 10]. We show that caspase-8 is still capable of mediating death even in the absence of this particular autocleavage, suggesting that caspase-8 participates in inflammasome-mediated cell death and does not induce apoptosis during *Legionella* infection.

Minor Comment #1: *The sentence: “Using this priming and infection model alongside genetic tools, our data demonstrate that TNF signaling through TNFR1 licenses L. pneumophila-infected cells to rapidly and robustly undergo flagellin and NAIP/NLRC4-independent cell death in a T4SS-dependent manner” Is somewhat misleading, since it is known that T4SS is indispensable for Legionella replication under all circumstances.*

**Response:** We thank the reviewer for this suggestion and have subsequently modified this sentence for clarity.

Minor Comment #2: *“TNF is able to license this pyroptotic death in part by upregulating components of the caspase-11 non-canonical inflammasome ahead of infection” is inaccurate since the authors did not exclude other types of cell death such as necrosis and apoptosis (PANoptosis).*

**Response:** Our data show a requirement for caspases-1, -8, and -11 and the pyroptotic mediators gasdermin D and E in TNF-licensed cell death early during *L. pneumophila* infection. Neither RIPK3 (required for the execution of necroptosis) nor caspase-8 activity (required for classical extrinsic apoptosis) were required. We additionally observed no caspase-3/7 activity. Thus, we believe we have sufficiently excluded necroptosis and apoptosis , indicating that the death we observe is primarily occurring through pyroptosis. (Please see also response to Major Comment #1.)

Minor Comment #3: *Please make sure to add the appropriate references for the role of flagellin in Legionella replication and its recognition by Nlrc4/Ipaf.*

**Response:** We thank the reviewer for the suggestion and we have added the recommended references.

Minor Comment #4: *Please discuss the findings in relation to other similar studies in the field such as Mascarenhas et al 2017, Santic ela al 2007, kawamoto et al 2017,*

**Response:** We thank the viewer for the suggestion We have added discussion of the recommended studies to the manuscript.

1.	DeLaney AA, Berry CT, Christian DA, Hart A, Bjanes E, Wynosky-Dolfi MA, et al. Caspase-8 promotes c-Rel-dependent inflammatory cytokine expression and resistance against Toxoplasma gondii. Proc Natl Acad Sci U S A. 2019;116(24):11926-35.

2.	Gitlin AD, Heger K, Schubert AF, Reja R, Yan D, Pham VC, et al. Integration of innate immune signalling by caspase-8 cleavage of N4BP1. Nature. 2020;587(7833):275-80.

3.	Case CL, Kohler LJ, Lima JB, Strowig T, de Zoete MR, Flavell RA, et al. Caspase-11 stimulates rapid flagellin-independent pyroptosis in response to Legionella pneumophila. Proc Natl Acad Sci U S A. 2013;110(5):1851-6.

4.	Casson CN, Copenhaver AM, Zwack EE, Nguyen HT, Strowig T, Javdan B, et al. Caspase-11 activation in response to bacterial secretion systems that access the host cytosol. PLoS Pathog. 2013;9(6):e1003400.

5.	Broz P, Monack DM. Noncanonical inflammasomes: caspase-11 activation and effector mechanisms. PLoS Pathog. 2013;9(2):e1003144.

6.	Kayagaki N, Stowe IB, Lee BL, O'Rourke K, Anderson K, Warming S, et al. Caspase-11 cleaves gasdermin D for non-canonical inflammasome signalling. Nature. 2015;526(7575):666-71.

7.	Lee BL, Stowe IB, Gupta A, Kornfeld OS, Roose-Girma M, Anderson K, et al. Caspase-11 auto-proteolysis is crucial for noncanonical inflammasome activation. J Exp Med. 2018;215(9):2279-88.

8.	Ross C, Chan AH, Von Pein J, Boucher D, Schroder K. Dimerization and auto-processing induce caspase-11 protease activation within the non-canonical inflammasome. Life Sci Alliance. 2018;1(6):e201800237.

9.	Philip NH, DeLaney A, Peterson LW, Santos-Marrero M, Grier JT, Sun Y, et al. Activity of Uncleaved Caspase-8 Controls Anti-bacterial Immune Defense and TLR-Induced Cytokine Production Independent of Cell Death. PLoS Pathog. 2016;12(10):e1005910.

10.	Tummers B, Mari L, Guy CS, Heckmann BL, Rodriguez DA, Ruhl S, et al. Caspase-8-Dependent Inflammatory Responses Are Controlled by Its Adaptor, FADD, and Necroptosis. Immunity. 2020;52(6):994-1006 e8.
